# Supplementary material for: Factors limiting reproductive success in urban Greylag Geese (Anser anser)
Source: PeerJ. 2022 Aug 4;10:e13685. doi: 10.7717/peerj.13685 (PMC9357368; doi:10.7717/peerj.13685)
Supplement: Supplemental Information 1 [file peerj-10-13685-s001.docx]

**Factors limiting reproductive success in urban Greylag Geese (*Anser anser*)**

**Supplemental Material S1**

**Table S1.** Yearly percentage of breeders and non-breeders in the local population during March and April (n, number of geese).

| **Year** | **% Breeder** | **% Non-breeder** | **n** |
| --- | --- | --- | --- |
| **2007** | 20% | 80% | 70 |
| **2008** | 16% | 84% | 77 |
| **2009** | 28% | 72% | 99 |
| **2010** | 27% | 73% | 127 |
| **2011** | 28% | 72% | 171 |
| **2012** | 17% | 83% | 180 |
| **2013** | 22% | 78% | 172 |
| **2014** | 19% | 81% | 190 |
| **2015** | 24% | 76% | 216 |
| **2016** | 17% | 83% | 200 |
| **2017** | 14% | 86% | 182 |
| **2018** | 14% | 86% | 216 |
| **2019** | 13% | 87% | 194 |
| **2020** | 15% | 85% | 184 |


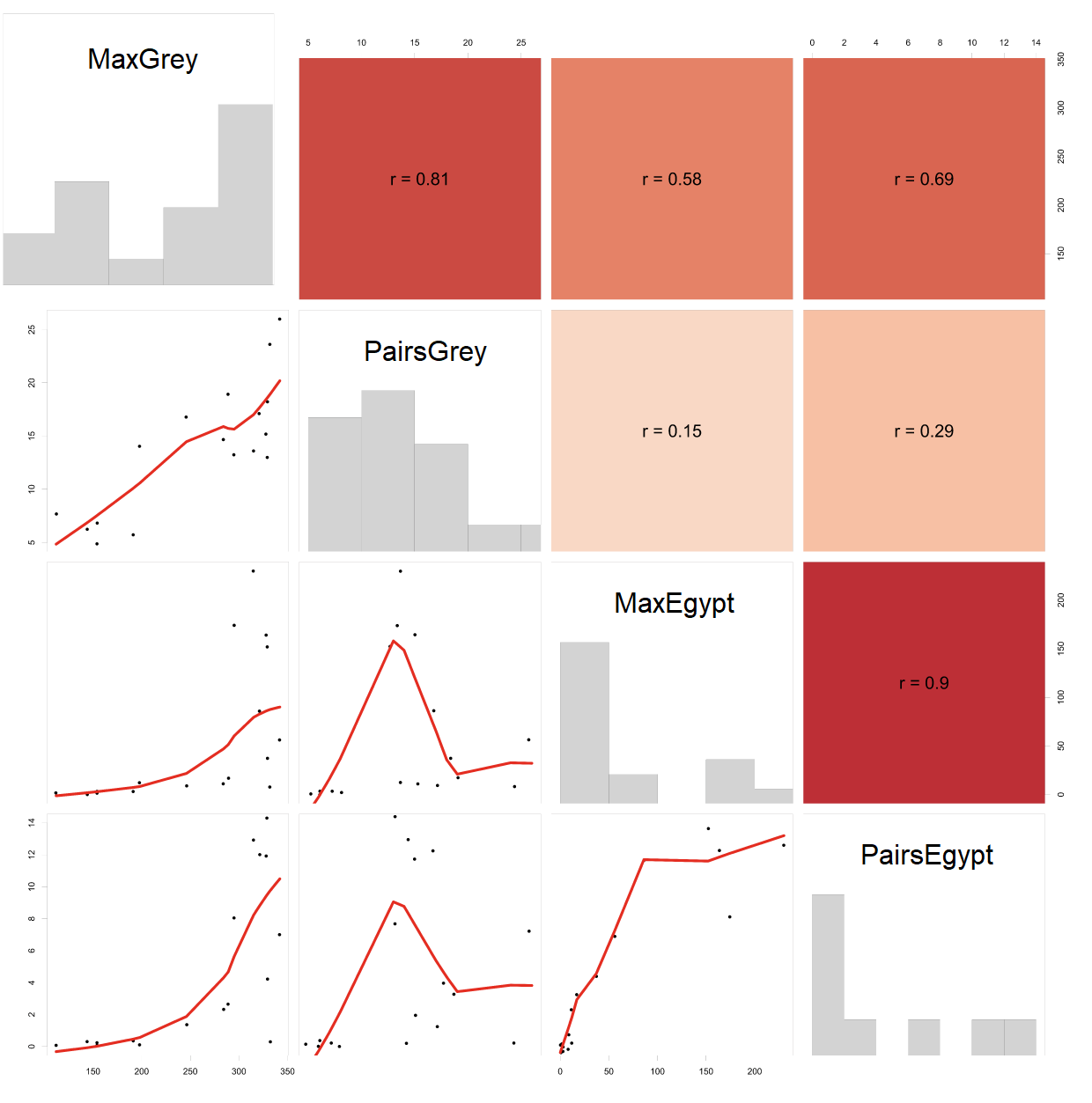


**Figure S1.** Correlation coefficients between the four variables MaxGrey (maximum count of Greylag Geese during moult), PairsGrey (number of Greylag Goose pairs), MaxEgypt (maximum count of Egyptian Geese in a year) and PairsEgypt (number of Egyptian Goose pairs).

**
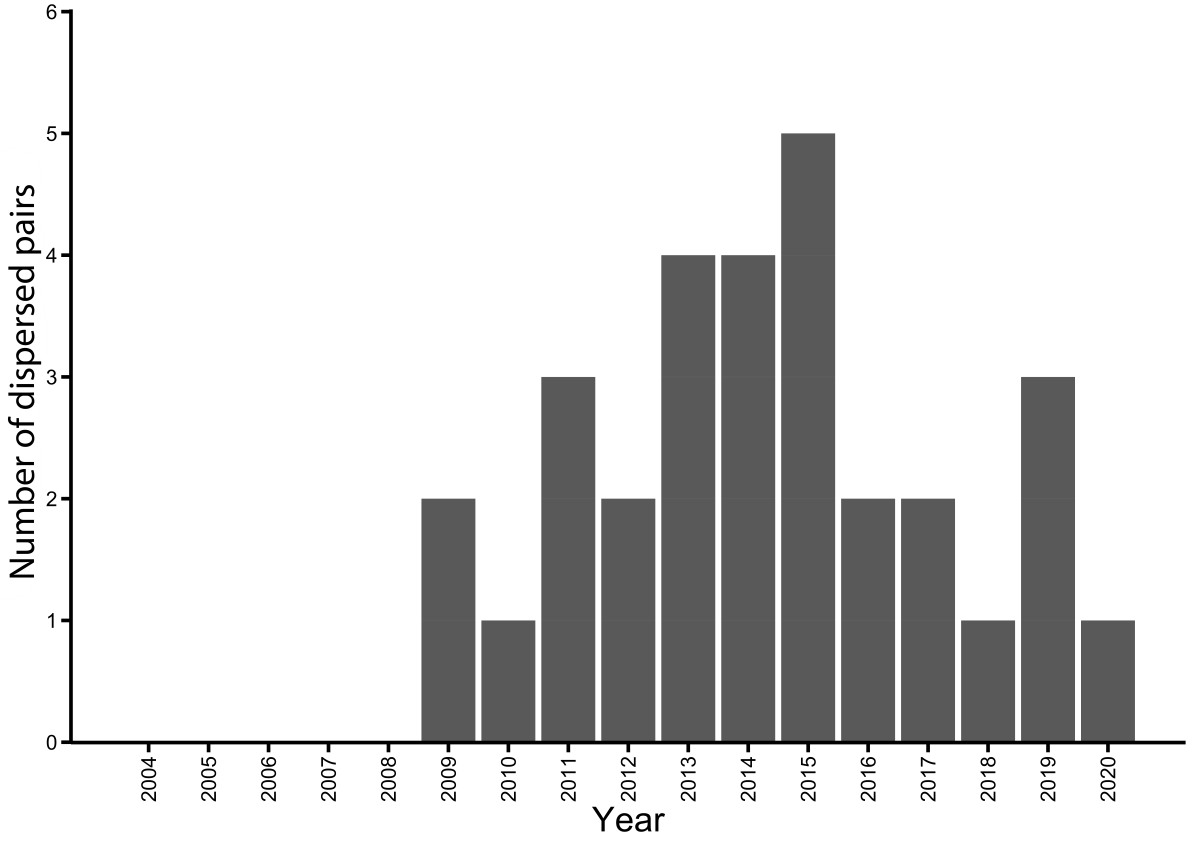
**

**Figure S2.** Number of dispering pairs per year. Some pairs dispersed in several years.

**Table S2.** Parameter estimates for the six models with the lowest AICc, which describe the effect of each explanatory variable in the various models on the probability of hatchling survival of Greylag Geese. Grey shaded models contain variables with strong collinearity. The last line shows the cumulative variable Akaike weight (*w_i_*) for each variable for all models (n = 2099). Greylag Geese: Dispersal, whether a pair dispersed with their young or not (yes/no); Hatchlings, number of hatchlings per brood; MaxGrey, maximum annual count during moult; PairsGrey, number of breeding pairs. Egyptian Geese: PairsEgypt, number of breeding pairs.

| **Model** | **Intercept** | **Dispersal** | **Hatch-lings** | **MaxGrey** | **Pairs Grey** | **Pairs Egypt** | **Dispersal* Hatchlings** | **Dispersal*MaxGrey** |
| --- | --- | --- | --- | --- | --- | --- | --- | --- |
| M1 | 0.55 | 0.69 | 0.19 | -0.65 |  |  | -0.30 |  |
| M2 | 0.53 | 0.68 | 0.19 |  | -0.50 | -0.41 | -0.31 |  |
| M3 | 0.53 | 0.64 | 0.20 | -0.62 |  |  | -0.28 | 0.11 |
| M4 | 0.54 | 0.65 | 0.27 | -0.64 |  |  |  |  |
| M5 | 0.53 | 0.69 | 0.19 | -0.55 | -0.14 |  | -0.30 |  |
| M6 | 0.55 | 0.68 | 0.19 | -0.59 |  | -0.11 | -0.31 |  |
| **Variable weight (*w_i_*)** | | 1 | 0.75 | 0.69 | 0.65 | 0.66 | 0.29 | 0.26 |
